# Body-Related Social Comparison and Disordered Eating among Adolescent Females with an Eating Disorder, Depressive Disorder, and Healthy Controls

**DOI:** 10.3390/nu4091260

**Published:** 2012-09-11

**Authors:** Andrea E. Hamel, Shannon L. Zaitsoff, Andrew Taylor, Rosanne Menna, Daniel Le Grange

**Affiliations:** 1 Department of Psychology, Simon Fraser University, 8888 University Drive, Burnaby, BC V5A 1S6, Canada; Email: ahamel@sfu.ca; 2 Windsor Essex Community Health Centre, 1585 Ouellette Ave, Windsor, ON N8X 1K5, Canada; Email: ataylor@wechc.org; 3 Department of Psychology, University of Windsor, 401 Sunset Avenue, Windsor, ON N9B 3P4, Canada; Email: rmenna@uwindsor.ca; 4 Department of Psychiatry, The University of Chicago, 5841 South Maryland Avenue, MC3077, Chicago, IL 60637, USA; Email: legrange@uchicago.edu

**Keywords:** adolescents, anorexia nervosa, bulimia nervosa, eating disorder, social comparison

## Abstract

The purpose of this study was to investigate the association between body-related social comparison (BRSC) and eating disorders (EDs) by: (a) comparing the degree of BRSC in adolescents with an ED, depressive disorder (DD), and no psychiatric history; and (b) investigating whether BRSC is associated with ED symptoms after controlling for symptoms of depression and self-esteem. Participants were 75 girls, aged 12–18 (25 per diagnostic group). To assess BRSC, participants reported on a 5-point Likert scale how often they compare their body to others’. Participants also completed a diagnostic interview, Eating Disorders Inventory-2 (EDI-2), Beck Depression Inventory-II (BDI-II), and Rosenberg Self-Esteem Scale (RSE). Compared to adolescents with a DD and healthy adolescents, adolescents with an ED engaged in significantly more BRSC (*p* ≤ 0.001). Collapsing across groups, BRSC was significantly positively correlated with ED symptoms (*p* ≤ 0.01), and these associations remained even after controlling for two robust predictors of both ED symptoms and social comparison, namely BDI-II and RSE. In conclusion, BRSC seems to be strongly related to EDs. Treatment for adolescents with an ED may focus on reducing BRSC.

## 1. Introduction

Eating disorders affect approximately five percent of adolescent females and are associated with malnutrition, osteoporosis, depression, and in some cases, death [1,2]. Given the health risks associated with eating disorders, it is important for researchers and health care providers to understand the factors associated with the etiology and maintenance of disordered eating. One theory which has been proposed to explain the etiology and maintenance of eating disorders is social comparison theory. According to social comparison theory, humans have a need to assess their personal characteristics and abilities, and they do so by comparing themselves to others [3]. When an unfavorable discrepancy is perceived between self and other, the individual is motivated to adjust his or her behavior in order to minimize the discrepancy [4].

Researchers have hypothesized that eating disorder symptoms may result from maladaptive social comparisons related to appearance. That is, females who compare their appearance to that of others too frequently are likely to experience body dissatisfaction and disordered eating. In support of this hypothesis, Tiggemann [5] has found that adolescent boys and girls who reported spending more time watching television programs featuring attractive actors and actresses demonstrated an increased desire to be thin, purportedly due to an increased level of social comparison to attractive media icons. Other studies have revealed similar findings [6,7]. Although these correlational studies cannot determine whether media exposure *causes* eating disorder symptoms, a meta-analysis on experimental studies confirmed that exposure to media-portrayed thin-ideal images in the laboratory results in acute increases in body dissatisfaction [8].

More recent research suggests that media icons are not the only influential source of social comparison for adolescents. Using a large, nationally representative American sample, Mueller and colleagues [9] found that teens are also influenced by the social comparisons they make to their peers. More specifically, these researchers found that adolescent girls were more likely to report weight loss behaviors if the proportion of underweight females in their school was relatively high. However, the strongest predictor of weight loss behavior in this study was the weight loss behavior of *similar* peers; that is, girls were *especially *likely to report weight loss behaviors if a relatively high proportion of girls *with a similar BMI* were also engaging in weight loss behaviors. Thus, according to this study, the weight loss behaviors of teenage girls are influenced not only by thin-ideal images (in the media *and* in their school) but also by the weight loss behaviors of peers who are similar in body size.

The aforementioned studies do not measure social comparison directly but *assume* social comparison via exposure to certain images/models. However, further support for the role of social comparison in disordered eating comes from studies which measure social comparison directly (*i.e*., through questionnaires). Overall, these studies show that disordered eating is associated with higher levels of both *general* and *appearance-related* social comparison in undergraduate populations [10,11,12,13,14]. A recent meta-analysis summarized the findings from 156 studies that focused on the relationship between body dissatisfaction (a major risk factor for disordered eating) and social comparison. This meta-analysis confirmed that appearance-related social comparison, whether induced in the laboratory or measured via questionnaire, is related to body dissatisfaction in both adolescents and adults [15].

To date, the vast majority of studies examining the relationship between social comparison and eating disturbances have been conducted on healthy undergraduate populations [15]. Very few studies have included a clinical sample, and those that *have* focused almost exclusively on adults [16,17,18]. To our knowledge, only two studies to date have examined social comparison in a clinical sample of adolescents [19,20]. Both of these studies showed that, in comparison to healthy adolescents, social comparison is more prevalent among adolescents with an eating disorder. However, neither study controlled for depressive symptoms or self-esteem, two variables which have commonly been found to be associated with both eating disorder symptoms and maladaptive social comparison [2,21,22,23]. Thus, it is unclear whether depressive symptoms or self-esteem may account for the relationship between social comparison and disordered eating. Also, neither study included a psychiatric control group. Therefore, it is unclear whether social comparison is related to disordered eating specifically or psychopathology more generally.

The purpose of this study was twofold. Our first aim was to investigate the hypothesis that adolescents with an eating disorder (ED) will engage in significantly more body-related social comparison (BRSC) than adolescents in a control group and adolescents in a psychiatric comparison group (*i.e*., adolescents with a depressive disorder). Since ED symptoms occur along a continuum from mild to severe, our second aim was to investigate: (a) whether BRSC is correlated with ED symptoms; and (b) whether this association holds after two related variables—namely self-esteem and depressive symptoms—have been accounted for. We chose to recruit adolescents for this study for a variety of reasons including the following: (a) adolescence is a time of increased susceptibility for the development of an eating disorder; (b) physical appearance is a particularly salient concern among adolescents; (c) research with healthy populations suggests that the association between social comparison and disordered eating is especially strong among adolescents; and (d) most of the literature on social comparison and disordered eating has focused on adults. We chose adolescents with a depressive disorder as a psychiatric comparison group because: (a) levels of comorbidity between ED and DD diagnoses are high; and (b) like ED populations, DD populations have been shown to demonstrate high levels of social comparison [22]. 

## 2. Methods

### 2.1. Participants

Data for this study was collected as part of a larger project on peer relationships in adolescents with eating disorders [24]. Participants included 75 females, aged 12 to 18 (*M* = 15.35, *SD* = 1.75). Twenty-five reported no psychiatric history (*i.e*., healthy control group); 25 met Diagnostic and Statistical Manual of Mental Disorders-IV-TR criteria for a depressive disorder (major depressive disorder, *n* = 20; dysthymia, *n* = 4) [25]; and 25 met DSM-IV-TR diagnostic criteria for an eating disorder [26]. Within the ED group, seventeen participants presented with anorexic symptoms. Among these participants, nine met full DSM-IV-TR criteria for AN and eight met subthreshold criteria (*i.e*., met all the criteria for AN except the weight loss and/or amenorrhea criteria). Among the eight that met subthreshold criteria for AN, one did not meet the weight loss criterium, two did not meet the amenorrhea criterium, and five did not meet either the weight loss or amenorrhea criteria. Eight participants in the ED group presented with bulimic symptoms. Among these participants, four met full DSM-IV-TR criteria for BN, and four met subthreshold criteria (*i.e*., met all the criteria for BN except the frequency or duration of binge/purge behavior). Finally, fourteen participants (56 percent) in the ED group also met DSM-IV-TR criteria for a depressive disorder (twelve for major depressive disorder, and two for dysthymia). 

In terms of ethnicity, 47 participants (62.7%) were Caucasian; 14 (18.7%) were Black; 5 (6.7%) were Hispanic; 2 (2.7%) were Asian/Pacific; and 7 (9.3%) were of a different ethnicity. Because most adolescents who present for ED treatment are female [26], all participants in this study are female.

### 2.2. Procedure

Participants with an ED or DD were recruited from outpatient mental health clinics. Participants in the healthy control group were recruited from an adolescent community-center. Written assent and consent was obtained from participants and parents/guardians, respectively. 

Procedures were approved by institutional review boards at each of the mental health clinics including the ethics committee at the University of Windsor. Participants in the ED and DD group completed a semi-structured diagnostic interview and questionnaires assessing demographic information, eating disorder and depressive symptoms, self-esteem, and BRSC. Diagnostic interviews were conducted by two advanced clinical psychology graduate students and were confirmed by a registered psychologist. Participants in the healthy control group also completed these questionnaires, but instead of a semi-structured diagnostic interview, completed a screening measure for mental health problems. Those participants in the control group who indicated current symptoms on the screening measure were asked follow-up questions to assess symptom severity and completed a diagnostic interview as necessary. Those participants who met diagnostic criteria for an ED (*n* = 1) or DD (*n* = 2) were moved to the appropriate clinical group. Those who demonstrated symptoms of an ED or DD but did not meet full diagnostic criteria were excluded (*n* = 1). In total, four adolescents were excluded—one in the ED group who did not meet DSM-IV-TR criteria for an ED and three in the control group who reported subdiagnostic mental health problems. As a result of this procedure, none of the participants in the DD group or control group demonstrated eating disorder symptoms; however, participants in the ED group with depressive symptoms were not excluded, and therefore, some of these participants (*n* = 14, 56 percent) have comorbid ED–DD diagnoses. 

### 2.3. Measures

*Demographic Information*. Participants’ age, height, weight, ethnicity, parental education level, and parental relationship status (e.g., married, divorced, *etc*.) were assessed in a demographic questionnaire. BMI *z*-scores were calculated using age, weight, and height measurements and were based on Centers for Disease Control and Prevention (CDC) 2000 data [27]. To assess pubertal status, participants reported whether or not they had experienced menarche. 

*Kiddie-Schedule for Affective Disorders and Schizophrenia—Present Version *(M-KSADS-P) [28]. The KSADS-P was used to diagnose current Axis-I DSM-IV disorders. The KSADS-P is a semi-structured interview with high inter-rater reliability and good predictive and concurrent validity with adolescents [29,30,31].

*Body-Related Social Comparison*. BRSC was measured with a single question modeled after the items within the Body Comparison Scale (BCS) [32]. The BCS is a 36-item questionnaire which assesses the frequency with which respondents compare their body to that of others. For the purpose of keeping the large battery of assessments for this study as short as possible, and given the high internal consistency of the BCS subscales, a single composite item was formulated from the items within the General Body Comparison subscale of the BCS [33]. Specifically, participants were asked to report how often they compare their body to that of others. As in the BCS, responses were made on a 5-point Likert scale ranging from 1 (“Never”) to 5 (“Always”).

*Eating Disorders Inventory-2* (EDI-2) [34]. The EDI-2 is a 91-item self-report questionnaire assessing cognitive, emotional, and behavioral symptoms of eating disorders. The EDI-2 has high test-retest reliability (*r* = 0.75 to 0.94), good internal consistency (Cronbach’s α = 0.73 to 93), and has been used extensively with adolescents [35,36]. Three subscales of the EDI-2 were used in this study: Drive for Thinness (DT), Bulimia (B), and Body Dissatisfaction (BD). 

*Beck Depression Inventory—21-Item Version* (BDI-II) [37]. The BDI-II is a self-report questionnaire which assesses cognitive, emotional, and behavioral symptoms of depression. It has been used extensively with adolescents and has good internal consistency and test-retest reliability [38,39].

*Rosenberg Self-Esteem Scale *(RSE) [40]*. *The RSE is a 10-item self-report questionnaire used to assess global feelings of self-esteem. It has high test-retest reliability and strong construct, convergent, and discriminant validity [41,42,43,44]. Participants were asked to make their responses on a 5-point Likert scale ranging from 1 (“Not at all descriptive of me”) to 5 (“Very descriptive of me”).

### 2.4. Data Analysis

A one-way analysis of variance (ANOVA) was performed to compare groups (*i.e*., ED, DD, and healthy control) on BRSC. Tukey’s Honestly Significant Difference (HSD) *post-hoc* pairwise comparisons were used to determine which groups were significantly different on BRSC. Second, collapsing across diagnostic groups, a correlation matrix of all study variables was obtained to investigate the zero-order correlations between BRSC and ED symptoms. Finally, and again collapsing across groups, three multiple regression analyses were performed (one on each of the three EDI-2 subscales) to determine whether BRSC was a significant predictor of ED symptoms even after controlling for RSE and BDI-II scores.

## 3. Results

*Descriptive Statistics*. The majority of participants had parents who were married (64.0%) and who had at least some post-secondary education (68.1% for fathers and 69.8% for mothers). Most participants (94.7%) reported having experienced menarche. Study groups (*i.e*., ED, DD, and control) did not differ in terms of age, pubertal status, parental relationship status, mother’s level of education, or father’s level of education. Within the ED group, participants with anorexia and bulimia did not differ from each other in terms of RSE scores (*t*(22) = 0.42, *p* > 0.65), BDI-II scores (*t*(23) = −0.93, *p* > 0.35), or BRSC (*t*(23) = −1.07, *p* > 0.25). Thus, these participants were kept in a single group (*i.e*., ED group) for analyses. 

Ethnicity differed across the three study groups (χ^2^(8, *n* = 75) = 31.00, *p* ≤ 0.001). Within the ED group, the majority of adolescents (*n* = 20, 80%) were Caucasian, 4 (16%) were Hispanic, and 1 (4%) identified herself as “other”. Within the DD group, the majority of adolescents (*n* = 12, 48%) were Caucasian, 10 (40%) were African American, 2 (8%) were Asian, and 1 (4%) was Hispanic. Finally, within the healthy control group, the majority of adolescents (*n* = 15, 60%) were Caucasian, 6 (24%) identified themselves as “other”, and 4 (16%) were African American. Although ethnicity differed across groups, results of this study are unlikely to be confounded by ethnicity. ANOVA analyses revealed that ethnicity did not have an effect on BRSC (*p* > 0.15) or any of the EDI-2 subscales (*p* > 0.15). 

Table 1 displays descriptive statistics and ANOVA results for BMI *z-*scores, EDI-2 scores, BDI-II scores, and RSE scores across diagnostic groups. As expected, an ANOVA on BMI *z-*scores was significant (*F*(2, 69) = 12.30, *p* ≤ 0.001, η*_p_*^2^ = 0.26). Tukey’s HSD *post-hoc* comparisons revealed that adolescents in the ED group had significantly lower BMI *z*-scores than adolescents in the DD group (*p* ≤ 0.001) and healthy control group (*p* ≤ 0.05). Also as expected, a Multivariate ANOVA on EDI-2 scores was significant (*F*(2, 72) = 6.152, *p* ≤ 0.001, η*_p_*^2^ = 0.21). *Post-hoc* comparisons revealed that adolescents in the ED group had significantly higher scores on Drive for Thinness and Bulimia than adolescents in the healthy control (*p* ≤ 0.001 for both DT and B) and DD groups (*p* ≤ 0.001 for DT, *p* ≤ 0.05 for B). In regards to Body Dissatisfaction, adolescents in the ED group had significantly higher scores than adolescents in the healthy control group (*p* ≤ 0.001) only; Body Dissatisfaction scores did not differ across the ED and DD groups. Also as expected, an ANOVA revealed that BDI-II scores differed across groups (*F*(2, 72) = 14.80, *p* ≤ 0.001, η*_p_*^2^ = 0.29). *Post-hoc* comparisons revealed that adolescents in both the ED and DD group had significantly greater BDI-II scores than adolescents in the healthy control group (*p* ≤ 0.001 for both comparisons). BDI-II scores did not differ across the ED and DD groups. Finally, and also as expected, an ANOVA revealed that RSE scores differed across groups (*F*(2, 70) = 11.62, *p *≤ 0.001, η*_p_*^2^ = 0.25). *Post-hoc* comparisons revealed that adolescents in both the ED and DD group displayed significantly greater RSE scores than adolescents in the healthy control group (*p* ≤ 0.001 for both comparisons). 

**Table 1:** Descriptive Statistics and ANOVA Results for Body Mass Index, Eating Disorder Inventory-2 scores, Beck Depression Inventory-II scores, and Rosenberg Self-Esteem Scale Scores.

Variable		ED	DD	Control	*F*	η*_p_*^2^
		*M* (*SD*)	*M* (*SD*)	*M* (*SD*)		
BMI		−0.39 (1.17)	1.12 (0.93)	0.40 (1.03)	12.30 *	0.26
EDI-2					6.15 *	0.21
	EDI-DT	11.80 (5.16)	6.80 (4.72)	4.44 (3.27)		
	EDI-B	4.92 (5.08)	2.28 (2.37)	1.28 (1.84)		
	EDI-BD	14.72 (6.79)	11.48 (6.15)	7.48 (5.52)		
BDI-II		22.72 (14.80)	21.08 (12.48)	6.76 (3.96)	14.80 *	0.29
RSE		30.00 (10.72)	31.88 (8.89)	41.42 (6.24)	11.62 *	0.25

Note: BMI = Body Mass Index *z*-scores; EDI-2 = Eating Disorders Inventory-2; DT = drive for thinness; B = bulimia; BD = body dissatisfaction; BDI = Beck Depression Inventory II; ED = Eating Disorder Group; DD = Depressive Disorder Group; RSE = Rosenberg Self-Esteem Scale. *F* value for EDI is from a Multivariate ANOVA analysis on EDI-DT, EDI-B, and EDI-BD. * *p *≤ 0.001.

*Body-Related Social Comparison*. As hypothesized, groups differed in terms of BRSC (*F*(2, 71) = 8.23, *p* ≤ 0.001, η*_p_*^2^ = 0.19). Tukey’s HSD *post-hoc* comparisons revealed that participants in the ED group (*M* = 4.20, 95% CI [3.80, 4.60], range = 3) reported significantly higher BRSC scores than participants in the DD group (*M* = 3.44, 95% CI [2.92, 3.96], range = 4; *p *≤ 0.05) and healthy control group (*M* = 3.00, 95% CI [2.63, 3.37], range = 4; *p *≤ 0.001). No significant difference in BRSC was found between the DD and healthy control group. 

Table 2 displays correlations among study variables (*i.e*., BRSC, EDI-DT, EDI-B, EDI-BD, BDI-II, and RSE). As shown, BRSC is significantly positively correlated with scores on the EDI-DT (*r* = 0.64, *p* ≤ 0.01), EDI-B (*r* = 0.42, *p* ≤ 0.01), and EDI-BD (*r* = 0.66, *p* ≤ 0.01). 

**Table 2:** Correlation Matrix for Study Variables.

	BRSC	EDI-DT	EDI-B	EDI-BD	BDI-II	RSE
BRSC	--	64 *	42 *	66 *	50 *	−0.44 *
EDI-DT		--	49 *	69 *	53 *	−0.55 *
EDI-B			--	42 *	42 *	−0.37 *
EDI-BD				--	55 *	−0.51 *
BDI-II					--	−0.80 *
RSE						

Note: BRSC = body-related social comparison; EDI = Eating Disorders Inventory-2; DT = drive for thinness; B = bulimia; BD = body dissatisfaction; BDI = Beck Depression Inventory II; RSE = Rosenberg Self-Esteem Scale. * *p* ≤ 0.01.

Table 3 displays the results of the regression analyses on each of the three EDI-2 subscales. These regression analyses revealed that BRSC was a significant predictor of Drive for Thinness (β = 0.48, *p* ≤ 0.001), Bulimia (β = 0.29, *p* ≤ 0.05), and Body Dissatisfaction (β = 0.54, *p* ≤ 0.001), even after controlling for RSE and BDI-II scores. Over and above the variance predicted by RSE and BDI-II scores, BRSC predicted an additional 17% (*p* ≤ 0.001), 6% (*p* ≤ 0.05), and 21% (*p* ≤ 0.001) of the variance in Drive for Thinness, Bulimia, and Body Dissatisfaction, respectively. 

**Table 3:** Summary of Three Separate Hierarchical Regression Analyses for the Prediction of Scores on EDI-2 Subscales.

Variable		*B*	*SE* (*B*)	β	*t*	Δ*R*^2^
**Regression 1: EDI-Drive for Thinness**						
Step 1						0.31 ***
	RSE	−0.18	0.09	−0.33	−1.98	
	BDI-II	0.10	0.07	0.26	1.56	
Step 2			0.08			0.17 ***
	RSE	−0.15	0.06	−0.29	−1.97	
	BDI-II	0.02	0.47	0.05	0.34	
	BRSC	2.25		0.48	4.80 ***	
**Regression 2: EDI-Bulimia**						
Step 1						0.17 **
	RSE	−0.04	0.07	−0.09	−0.51	
	BDI-II	0.09	0.05	0.33	1.82	
Step 2						0.06 *
	RSE	−0.03	0.07	−0.07	−0.38	
	BDI-II	0.06	0.05	0.21	1.13	
	BRSC	0.94	0.40	0.29	2.33 *	
**Regression 3: EDI-Body Dissatisfaction**						
Step 1						0.30 ***
	RSE	−0.12	0.12	−0.18	−1.05	
	BDI-II	0.20	0.09	0.40	2.37 *	
Step 2						0.21 ***
	RSE	−0.09	0.10	0.13	−0.90	
	BDI-II	0.09	0.07	0.17	1.14	
	BRSC	3.19	0.58	0.54	5.49 ***	

Note: EDI = Eating Disorders Inventory-2; RSE = Rosenberg Self-Esteem Scale; BDI-II = Beck Depression Inventory-II; Δ*R*^2^ = change in *R*^2^. * p < 0.05, ** *p *< 0.01, *** *p *< 0.001.

## 4. Discussion

Results of this study support the hypothesis that maladaptive BRSC is strongly related to disordered eating. Adolescents in the ED group engaged in significantly more BRSC than adolescents in both the healthy control and DD group. Furthermore, BRSC was a significant predictor of ED symptoms, even after controlling for two robust predictors of both ED symptoms and social comparison—self-esteem and depressive symptoms. Taken together, these results suggest that BRSC may be particularly relevant to eating disorders rather than common to other forms of psychopathology like depressive disorders. 

The relationship between BRSC and disordered eating appears to be a strong one. After controlling for self-esteem and depressive symptoms, BRSC accounted for an additional 17 and 21 percent of the variance in drive for thinness (*i.e*., desire to be thin; EDI-DT) and body dissatisfaction (EDI-BD), respectively. These figures constitute medium to large effect sizes according to Cohen’s conventions [45]. The relationship between BRSC and bulimic symptoms appears to be somewhat weaker (albeit still significant), with BRSC accounting for an additional 6 percent of the variance in bulimic symptoms (a small to medium effect size). The strong association between BRSC and (a) drive for thinness; and (b) body dissatisfaction may be explained in part by the fact that like BRSC, drive for thinness and body dissatisfaction represent constructs which are cognitive in nature. These two constructs are measured in the EDI-2 with items such as “I am terrified of gaining weight.” and “I think that my stomach is too big.” Bulimia, on the other hand, represents a construct that is more behavioral in nature, measured in the EDI-2 with items such as “I stuff myself with food.” 

This study had a number of limitations which should be addressed in future research. First, BRSC was measured using only a single question regarding how frequently adolescents compare their body to that of others. However, this question resembles the types of questions appearing in social comparison measures which have been commonly used in this literature (e.g., Body Comparison Scale, Social Comparison to Models and Peers Scale) [31,46]. Nevertheless, future studies utilizing a recently developed and validated measure of BRSC (*i.e*., the Upward and Downward Physical Appearance Comparison scale) might provide a more detailed and reliable picture of the relationship between BRSC and disordered eating [47]. Second, the ED group in this study was heterogenous, with 56 percent of participants demonstrating a comorbid diagnosis of depression or dysthymia. This high rate of comorbidity makes it difficult (a) to discern what type of ED patient (*i.e*., ED-only or comorbid DD diagnosis) these results generalize to; and (b) to compare the effects of BRSC on an ED versus DD diagnosis. It should be noted, however, that many studies have documented similarly high rates of comorbidity between ED and DD diagnoses [48,49], making our ED sample representative of other ED populations.

A final limitation of this study was that it utilized a cross-sectional design, and therefore, conclusions regarding the direction of causality are precluded. It is the assumption of the authors that increased BRSC leads to an increased risk of developing ED symptoms. However, it is equally possible that ED symptoms (such as body dissatisfaction) may increase an individual’s tendency to compare their body to that of others, or that a third variable leads to both BRSC and ED symptoms. Given the cross-sectional nature of this study as well as the strength of the association between BRSC and ED, future studies should employ longitudinal analyses to determine whether or not BRSC represents a causal factor for ED symptoms. 

Despite these limitations, this study had a number of strengths. Namely, it is one of only three studies to explore the relationship between BRSC and disordered eating in a clinical sample of adolescents and the *only* study to utilize a psychiatric control group. Furthermore, this study controlled for two robust predictors of ED symptoms—self-esteem and depressive symptoms. These two variables have been associated with ED symptoms extensively and have been associated with social comparison as well [22,50]. Thus, this research provides a step toward establishing the specificity of BRSC as a risk factor for eating disorders.

## 5. Conclusions

In conclusion, results of this study have indicated that BRSC is strongly associated with EDs. Not only was BRSC more prevalent among adolescents with an ED, it was also strongly associated with ED symptoms—even after two robust predictors of ED symptoms (namely low self-esteem and depressive symptoms) had been accounted for. 

## 6. Implications

Results of this study provide implications for clinical practice. Because BRSC is so strongly related to eating disorder symptoms, especially cognitive aspects of the disorder, treatment and prevention programs for adolescents with an eating disorder may benefit from a focus on reducing BRSC. For example, adolescent patients should be encouraged to evaluate their body shape based on what is healthy rather than on what other people’s bodies look like. Similarly, both treatment and prevention programs may focus on debunking myths about the female (and male) body in order to minimize comparisons to attractive idols. For example, teens may be taught that the body shape and BMI of attractive models and actresses (and actors) is not representative of the average population and is often achieved in unhealthy ways. Similarly, teens may be taught that many magazine photos are digitally altered to make female models look more thin and attractive (and male models more muscular), and therefore, comparing ones body to magazine images may be a misleading and detrimental activity.

Given the importance of appearance as well as the importance of peers and celebrity icons during adolescence, reducing BRSC in adolescent eating disorder patients will likely prove a difficult task. Future studies should focus on ways in which eating disorder treatments may be modified to reduce BRSC in this population of patients.
